# Bronchial Responsiveness in Patients with Restrictive Spirometry

**DOI:** 10.1155/2013/498205

**Published:** 2013-08-18

**Authors:** Jean I. Keddissi, Marwan K. Elya, Saif U. Farooq, Houssein A. Youness, Kellie R. Jones, Ahmed Awab, Gary T. Kinasewitz

**Affiliations:** Division of Pulmonary/Critical Care Medicine, the Oklahoma City VA Medical Center and the University of Oklahoma Health Sciences Center, 920 Stanton L. Young Boulevard, WP 1310, Oklahoma City, OK 73104-5020, USA

## Abstract

*Background*. Improvement in PFT after bronchodilators is characteristic of obstructive airway diseases such as COPD. However, improvement in patients with restrictive pattern is occasionally seen. We aim to determine the clinical significance of a bronchodilator responsive restrictive defect. *Methods*. Patients with restrictive spirometry and a bronchodilator study were identified at the University of Oklahoma and Oklahoma City VAMC between September 2003 and December 2009. Restriction was defined as a decreased FVC and FEV1, with normal FEV1/FVC. Responsiveness to bronchodilators was defined as an improvement in FEV1 and/or FVC of at least 12% and 200 mL. Patients with lung volume measurements had their clinical and radiographic records reviewed. *Results*. Twenty-one patients were included in the study. Most were current or ex-smokers, with most being on bronchodilators. The average FVC and FEV1 were 65 ± 11% and 62 ± 10% of the predicted, respectively. Most patients (66%) had a normal TLC, averaging 90 ± 16% of the predicted. RV, RV/TLC, and the TLC-VA values strongly suggested an obstructive defect. *Conclusions*. Reversible restrictive pattern on spirometry appears to be a variant of obstructive lung disease in which early airway closure results in air trapping and low FVC. In symptomatic patients, a therapeutic trial of bronchodilators may be beneficial.

## 1. Introduction 

The use of short-acting bronchodilators, such as albuterol, during pulmonary function tests (PFTs) is a very common, routine test. Bronchial responsiveness to bronchodilators is a physiological response involving airway epithelium, nerves, mediators, and bronchial smooth muscles [1]. Based on the ATS guidelines, such responsiveness is considered to be present when the improvement in forced expiratory volume in 1 second (FEV1) or in the forced vital capacity (FVC) is at least 12% and 200 mL [1]. Increments of less than 8% are likely to be within measurement variability. 

The test has diagnostic, therapeutic, and prognostic implications in patients with obstructive disease [2, 3], such as asthma and chronic obstructive pulmonary disease (COPD). Its significance, when positive in a nonobstructive case (i.e., normal or restrictive spirometry) remains to be determined. 

While several case reports have described individual patients with a reversible restrictive lung patterns [4–8], the significance of a positive bronchial responsiveness test in a group of patients with purely restrictive spirometry has not been examined. Our aim was to evaluate the clinical characteristics of patients with restrictive spirometry and a significant response to bronchodilators and to distinguish between restrictive and obstructive disease based on their radiographic and lung volume findings. Our hypothesis is that bronchodilator responsiveness in patients with restrictive defect is likely indicative of an underlying obstructive airway disease. 

## 2. Methods 

This was a retrospective cohort study conducted at the University of Oklahoma and the Oklahoma City Veterans Affairs Medical Center. None of the authors has any conflict of interest associated with the study. After obtaining IRB approval to conduct the study (IRB number 13098), all PFTs meeting ATS criteria for acceptability and reproducibility [9], performed between September 2003 and December 2009 on patients older than 18, were reviewed. All patients with an initial restrictive spirometry, with significant response to bronchodilators who had pulmonary lung volumes measured, were included in our analysis.

All the studies were done by experienced pulmonary technologists, using SensorMedics Vmax 229D series (CareFusion, San Diego, CA, USA). Lung volumes were done on a V6200 AutoBox (CareFusion, San Diego, CA, USA). Normal spirometry values were calculated from the NHANES III database [10], while lung volumes and diffusion capacity (DLCO) normal values were derived from the studies by Crapo et al. [11, 12]. We defined a restrictive spirometry as one with a FVC <lower limit of normal (i.e., 5th percentile), with a normal FEV1/FVC. Normal FEV1/FVC was defined based on the NHANES III database and the ATS guidelines as values greater than 95% of the predicted for age, gender, and height [1, 10]. Positive response to bronchodilators was defined as a change in FEV1 or in the FVC of at least 12% and 200 mL [1]. Prebronchodilator maximum voluntary ventilation (MVV) was recorded, was considered decreased if it fell below the lower limit of normal, and was <32xFEV1 [9]. Lung volumes were measured using whole-body plethysmography. DLCO and alveolar volume (VA) were obtained by the single-breath method. Airway resistance (Raw) and specific conductance (sGaw) were measured and compared to reference values [13]. 

Medical records were reviewed. Patients characteristics, including demographics, smoking history, comorbidities, medication use, and symptoms at the time of the initial spirometry, were recorded. Radiographic findings on chest X-rays were also included. Data is expressed as mean ± standard deviation (SD). The percentage of patients with a decreased TLC (suggestive of a true restrictive defect) was calculated. 

## 3. Results 

Between September 2003 and December 2009, there were 7502 spirometries with bronchodilator evaluations performed and 1588 plethysmographic lung volume measurements, all done using whole-body plethysmography. During that period, we identified 21 patients with a reversible restrictive pattern on PFT, who had lung volumes measured. The average age was 65 years (range 48 to 81) (Table 1). Fifteen patients were male (71%), while the majority was Caucasian (86%). Only 6 patients (29%) never smoked. In those with a history of smoking, the average pack-year was 51 ± 29. The patients tended to be overweight, with a body mass index (BMI) of 31 ± 7 kg/m^2^ (range 23–47 kg/m^2^). 

Most patients were symptomatic (Table 2). Dyspnea was the main reason to obtain the PFT, followed by cough and wheezing. Only 1 patient did not have any of these complaints. Most patients did not show radiographic findings suggestive of a restrictive disease or of a chronic obstructive disease. More than half had a clinical history suggestive of asthma or COPD. One patient had a history of interstitial lung disease, while another two patients had a history of autoimmune disease without evidence of pulmonary involvement. The majority of patients were on inhaled bronchodilators and/or inhaled corticosteroids. 

Table 3 summarizes the baseline spirometry data. All patients had a restrictive defect on spirometry, with an average FEV1 of 62 ± 10% and FVC of 65 ± 11%. FEV1/FVC and FEV1/SVC were all normal. All patients had a significant response to bronchodilators (either by FEV1 or FVC criteria), with 10 patients having response to both. The MVV was reduced in 9 patients (43%). 

The total lung capacity (TLC) averaged 5.78 ± 1.44 L or 90 ± 16% of the predicted and was below the lower limit of normal in 7 patients (33%) (Table 4). In those 7 cases, the average TLC was 74% of the predicted. In 2 patients, the TLC was above the upper limit of normal. The average residual volume (RV) was high at 128 ± 38%. No RV fell below the lower limit of normal. The RV/TLC was elevated with a mean of 52 ± 9%. Airway resistance (Raw) was elevated in 16 patients (76%) with a mean of 253 ± 150%, while the average specific conductance was reduced at 42 ± 21%. 

The mean adjusted DLCO was 69 ± 24%. In 12 patients (57%), the DLCO was below the lower limit of normal. The average TLC-VA difference was 1.27 ± 0.90 L (range 0.33–3.54 L). 

## 4. Discussion 

Pulmonary function testing is an invaluable tool in the hands of pulmonologists and primary care physicians. It is used primarily as a diagnostic aide during the workup of respiratory symptoms, but PFTs are also helpful in the long-term patient monitoring as well as evaluation of potential disability and in epidemiological and research studies [9]. One of the first questions spirometry has to answer is whether an obstructive abnormality (defined as a reduced FEV1/VC below the 5th percentile of the predicted value) or a restrictive one (defined as reduced VC with normal or slightly increased FEV1/VC) is present [1]. Since the vital capacity may be diminished by both restrictive and obstructive ventilatory defects [14], a reduced VC does not prove the presence of a restrictive defects, and the presence of which has to be confirmed by low TLC. In fact, Aaron et al. showed that <60% of patients with a spirometric restrictive pattern (i.e., low FVC and normal or above normal FEV1/FVC) had pulmonary restriction confirmed on lung volume measurements [15]. Failure to exhale long enough to empty the lung to RV can result in a decreased FVC and FEV1 with a normal FEV1/FVC [1]. In addition, early airway closure with gas trapping may lead to airflow limitation at low lung volume, with incomplete lung emptying and hyperinflation [14]. This could be interpreted as a restrictive defect, when the true underlying pathology is an obstructive one. Slow VC may give a better estimate of the vital capacity, but measurement of lung volumes is usually needed to exclude restriction (normal TLC) and to show evidence of air trapping, as manifested by an increased RV and RV/TLC. 

A mixed pattern is defined as a FEV1/VC and a TLC that are both below the 5th percentiles of their predicted values. 

Bronchodilators during spirometry are typically used to assess the response of an obstructive defect. It is well recognized that the response could be seen in the FEV1, FVC, or both. Even though there is no consensus about what constitutes reversibility in patients with obstruction [16], the American Thoracic Society/European Respiratory Society defines significant response as >12% and 200 mL compared with baseline [1]. 

Our study is the first to systematically examine the clinical significance of a restrictive pattern (defined as a spirometry with decreased FVC, FEV1 with normal FEV1/FVC) that is responsive to bronchodilators in a group of patients referred for pulmonary function testing. The slow VC was measured at baseline in 13 patients. In all these cases the FEV1/SVC was within normal limits and therefore could not be used to infer the presence of an obstructive defect. Despite a spirometry that would be interpreted as showing moderate restriction, the TLC was normal in two-thirds of our patients, excluding significant restriction in these subjects [17]. None of our patients had a decreased RV as might be expected in a restrictive defect. In fact, the average RV/TLC was elevated at 52 ± 9%, with 10 patients (48%) having a ratio that was >95% of the predicted value for their age [18]. This would be expected to result from air trapping, a hallmark of obstructive lung diseases. In fact, most of our patients were current or ex-smokers, which puts them at risk for obstructive lung disease. In addition, the large difference between the TLC and alveolar volume (TLC-VA) points in the same direction and suggests maldistribution of ventilation as the result of an obstructive dysfunction. The elevated airway resistance with a reduced specific conductance is another typical finding of obstructive lung disease. Finally the measured DLCO was reduced in the majority of patients. Even though this is a feature of restrictive lung disease, it is also seen in obstructive pulmonary disease, such as emphysema [19]. 

Previous case reports have described individual patients with a reversible or bronchodilator responsive restrictive lung disease manifest by both a restrictive spirometric pattern and a reduced TLC. This reduction in lung volume has been attributed to the closure of the terminal lung units by contraction of alveolar ducts [5]. Miller and Palecki who reviewed the PFTs and medical records of 413 patients with a clinical diagnosis of asthma found that a restrictive pattern was relatively common and usually associated with an elevated BMI [20]. Pseudorestriction with air trapping was seen in only 7 patients. This is in contrast to the present study in which half of the patients had an elevated residual volume. 

The majority of patients in our study displayed the “nonspecific pattern” of pulmonary function test, in which FEV1 and FVC are reduced, while the FEV1/FVC and TLC are normal, described by Hyatt et al. [21]. This pattern was seen in approximately 10% of patients undergoing spirometry and lung volume measurements at the Mayo Clinic. A similar group of 12 patients was described in 2004 and were thought to suffer from small airway obstruction [22]. In a larger follow-up study from the Mayo Clinic group, a significant response to bronchodilators was seen in 11% of the patient with the nonspecific pattern [23]. While a response to bronchodilators, history of smoking, and large TLC-VA difference predicted the development of an obstructive defect, most patients continue to exhibit the same pattern on subsequent spirometry. This “small airways obstruction syndrome” is thought to be present in early emphysema, small airway disease, asthma, and in older age [24]. Even though a few our patients had a low TLC, the majority of our population are likely to be a different manifestation of the same phenomenon, where an obstructive defect does not present with the classical reduction of the FEV1/FVC. Rather, the clue to the presence of the obstructive defect is the response to bronchodilators. 

Due to the retrospective nature of our study, we cannot eliminate a selection or history bias. Lung volume measurements were not uniformly done in all patients with restrictive spirometry and bronchodilator response. Therefore, our group may not be truly representative of this condition. In addition, we cannot have a definite estimate of its true incidence. We only identified 21 patients who met these criteria, out of a total of 7502 studies suggesting that this is a rather rare event. However, since our group may have similar characteristics to the Mayo Clinic subgroup of patients with nonspecific pattern who were responsive to bronchodilators [21, 23], our best estimate from these 2 studies is that 1% of patients undergoing spirometry and lung volume may end up having this condition (11% response to bronchodilator among the 10% of patients in whom the FEV1 and FVC are reduced, while the FEV1/FVC and TLC are normal). Prospectively designed studies where all patients with this condition have measurements of their lung volumes will be needed to address these limitations. 

In summary, our report is the first to exclusively describe patients with a restrictive spirometry that is responsive to bronchodilators. In most cases, lung volume measurements exclude a significant restrictive defect and suggest the presence of an obstructive one. This is likely due to early airway closure resulting in air trapping. Clinicians need to be aware of this phenomena, and the possibility of underlying obstructive diseases needs to be strongly considered. Symptomatic patients may benefit from a therapeutic trial of bronchodilators. 

## Figures and Tables

**Table 1:** Demographics of the 21 patients with reversible restrictive spirometry.

Age (years)*	65 ± 9
Gender (M/F)	15/6
Race	
Caucasian	18 (86%)
African-American	3 (14%)
Smokers (*n*, %)	
Current	7 (33%)
Ex-smoker	8 (38%)
Never	6 (29%)
Pack-year*	51 ± 29
Body mass index (kg/m^2^)*	31 ± 7

*Data expressed as mean ± SD.

**Table 2:** Symptoms, radiographic findings, and medical history.

	*n* (%)
Symptoms	
Cough	13 (62%)
Dyspnea	18 (86%)
Wheezing*	7 (39%)
Radiographic findings^§^	
Fibrotic changes	2 (10%)
COPD changes	4 (20%)
Kyphosis/scoliosis	4 (20%)
Past medical history^¥^	
Asthma	6 (32%)
COPD	4 (21%)
Interstitial lung disease	1 (5%)
Chronic inflammatory/autoimmune disease	2 (11%)
Medications^¥^	
Bronchodilators	14 (74%)
Inhaled corticosteroids	10 (53%)
Systemic steroids	2 (11%)
Home O_2_	3 (16%)

*Wheezing data not available in 3/21 patients. ^§^CXR data not available in 1/21 patient. ^*¥*^Medication data not available in 2/21 patients.

**Table 3:** Baseline pulmonary function tests.

FEV1	
Observed, (L)	1.91 ± 0.58
% predicted	62 ± 10
<Lower limit of normal	20 (95)
Bronchodilator response (L)	0.32 ± 0.15
Bronchodilator response (%)	17.1 ± 6.3
FVC	
Observed, (L)	2.62 ± 0.82
% predicted	65 ± 11
<Lower limit of normal	21 (100)
Bronchodilator response (L)	0.41 ± 0.15
Bronchodilator response (%)	16.5 ± 6.1
FEV1/FVC	73 ± 5
FEV1/SVC	72 ± 5
MVV	
Observed, (L)	63 ± 23
% predicted	52 ± 16
<Lower limit of normal and <32 × FEV1	9 (43)

Prebronchodilators values are presented as mean ± SD or *n* (%).

**Table 4:** Lung volume measurements.

TLC	
Observed, (L)	5.78 ± 1.44
% predicted	90 ± 16
<Lower limit of normal	7 (33)
TLC-VA (L)	1.27 ± 0.90
VC	
Observed, (L)	2.75 ± 0.83
% predicted	68 ± 13
<Lower limit of normal	18 (86)
RV	
Observed, (L)	3.03 ± 0.96
% predicted	128 ± 38
<Lower limit of normal	0 (0)
RV/TLC	
Observed	0.52 ± 0.09
>Upper limit of normal	10 (48%)
Adjusted DLCO	
Observed, (mL/mmHg/min)	17.37 ± 6.97
% predicted	69 ± 24
<Lower limit of normal	12 (57)
Raw	
% predicted	253 ± 150
>Upper limit of normal	16 (76%)
sGaw	
% predicted	42 ± 21

Values are presented as mean ± SD or *n* (%). Raw: airway resistance; sGaw: specific conductance.
